# Response of Gut Microbiota to Metabolite Changes Induced by Endurance Exercise

**DOI:** 10.3389/fmicb.2018.00765

**Published:** 2018-04-20

**Authors:** Xia Zhao, Zhujun Zhang, Bin Hu, Wei Huang, Chao Yuan, Lingyun Zou

**Affiliations:** ^1^Bioinformatics Center, Department of Microbiology, Third Military Medical University, Chongqing, China; ^2^Department of Medical Laboratory Science, Southwest Hospital, Third Military Medical University, Chongqing, China; ^3^Center for Prenatal Diagnosis, Department of Obstetrics and Gynecology, Southwest Hospital, Third Military Medical University, Chongqing, China; ^4^Department of Stomatology, Southwest Hospital, Third Military Medical University, Chongqing, China; ^5^College of High Altitude Military Medicine, Third Military Medical University, Chongqing, China

**Keywords:** exercise, marathon, gut microbiota, microbiome, metabolites

## Abstract

A few animal studies have shown that wheel running could reverse an unhealthy status by shifting the gut microbial composition, but no investigations have studied the effect of endurance running, such as marathon running, on human gut microbial communities. Since many findings have shown that marathon running immediately causes metabolic changes in blood, urine, muscles and lymph that potentially impact the gut microbiota (GM) within several hours. Here, we investigated whether the GM immediately responds to the enteric changes in amateur half-marathon runners. Alterations in the metabolic profile and microbiota were investigated in fecal samples based on an untargeted metabolomics methodology and 16S rDNA sequencing analysis. A total of 40 fecal metabolites were found significantly changed after finishing a half-marathon race. The most significantly different metabolites were organic acids (the major increased metabolites) and nucleic acid components (the major decreased metabolites). The enteric changes induced by running did not affect the α-diversity of the GM, but the abundances of certain microbiota members were shown to be significantly different before and after running. The family *Coriobacteriaceae* was identified as a potential biomarker that links exercise with health improvement. Functional prediction showed a significantly activated “Cell motility” function of GM within participants after running. Correlation analysis indicated that the observed differential GM in our study might have been the shared outcome of running and diet. This study provided knowledge regarding the health impacts of marathon running from the perspective of GM for the first time. Our data indicated that long-distance endurance running can immediately cause striking metabolic changes in the gut environment. Gut microbes can rapidly respond to the altered fecal metabolites by adjusting certain bacterial taxa. These findings highlighted the health-promoting benefits of exercise from the perspective of GM.

## Introduction

Sedentary behavior is associated with an increased risk of multiple chronic illnesses and is considered an independent risk factor for both morbidity and mortality in recent epidemiologic findings (Warren et al., 2010). The prevalent sedentary lifestyle gives a strong reason for people to choose exercise as a healthy lifestyle. The beneficial effects of exercise have been well documented(Pedersen and Saltin, 2015). Regular running improves not only mental health but also physical condition, including the hematopoietic system, bone, muscle, immune system, and cardiovascular function (Warburton et al., 2006). Exercise is considered as a metabolism modulator. The metabolic changes, such as purine metabolites, tryptophan metabolites, as well as metabolites from the gut microbiota (GM), in blood, urine, and feces induced by running may be partially responsible for the running-related promotion of health (Daskalaki et al., 2015; Neufer et al., 2015). A previous research in rat observed that endurance training elevated the greater rate of tricarboxylic acid cycle and antioxidant activity (Huang et al., 2010). Since jogging does not require a professional training ground and skills, marathon running has become an increasingly popular form of athletics, especially among amateur runners (Traiperm et al., 2016). Previous studies suggested that hematological and biochemical markers changed immediately and returned to baseline levels after 2–7 days of recovery in amateur half-marathon runners. Some hormonal markers, such as insulin, leptin, and adiponectin, showed unique patterns after a marathon running (Arakawa et al., 2016; Bekos et al., 2016). Feces metabolomics revealed that exercise reduces the proportion of secondary bile acids in rats (Hagio et al., 2010). The repetitive mechanics of marathon running can promote intestinal motility and transit and thus reduce the transient stool time.

The health-promoting benefits of exercise have been recognized for thousands of years, yet the mechanisms by which exercise prevents disease and improves health outcomes are poorly understood. Several studies in animal models and humans have found correlations between specific alterations in the GM community structure and exercise. Animal studies demonstrated that wheel running exercise could reverse unhealthy states, such as diet-induced obesity, diabetes and toxicity, by shifting the gut microbial composition in mice (Choi et al., 2013; Evans et al., 2014; Lambert et al., 2015). Choi et al. (2013) reported that mice with running wheel exercise had an increase in phylum *Firmicutes* but decrease in phyla *Tenericutes* and *Bacteroidetes*, which attenuated changes in GM induced by oral exposure to polychlorinated biphenyls (PCB). Similarly, Lambert et al. (2015) described that exercised mice presented a greater abundance of *Firmicutes* species and lower *Bacteroides*/*Prevotella* genera compared with sedentary mice. By contrast, Evans et al. (2014) concluded that the exercise increased the *Bacteroidetes*, while decreased *Firmicutes* in mice. Two human studies of professional athletes suggested that exercise may increase the α-diversity of GM. Moreover, they found a significantly higher abundance of the bacterial genus *Akkermansia* and the enhancement of GM-derived SCFAs within the athletes (Clarke et al., 2014; Barton et al., 2017). However, no studies to date have provided information about whether an endurance (marathon running) exercise may influence the human GM.

The gastrointestinal tract provides a living environment for the GM, which performs nutritional, protective, and metabolic functions in the human body. A stable GM is critical in maintaining the health of humans. Although there is no gold standard to assess a “healthy” GM, alteration of the gut microbial composition and diversity may increase the risk of poor health conditions. Factors including age, genetics, diet, and lifestyle may influence the composition and metabolic functions of the microbiota (Faith et al., 2013). We hypothesize that metabolic changes in body fluids and high-impact repetitive mechanics in the gastrointestinal tract, caused by marathon running, may affect the intestinal environment and thereby influence the GM. The GM may be another mechanism by which marathon running exerts a positive effect on a healthy life. Identification of the microbiota-driven mechanisms underlying the link between exercise and improved health holds extraordinary promise for using microbiota intervention strategies in sports medicine.

Here, we investigated the fecal metabolites and microbiome in amateur runners before and after a half-marathon race using metabolomics and 16S rDNA sequencing analysis. The association of differential metabolites induced by running with the GM was further examined. This study is the first to reveal the health impacts of marathon running from the perspective of GM.

## Materials and Methods

### Study Design and Samples

This study recruited 20 health amateur runners (16 males and 4 females) who participated in the 2016 Chongqing International Half Marathon. Written informed consent was obtained from all the volunteers. This study was approved by the Ethics Board at the Third Military Medical University. A total of 40 fecal samples were collected before (BEF) and after (AFT) the half-marathon race at home with sterile sampling containers and immediately transported at 4°C to the laboratory. Each sample was subpacked in two 1.5 ml Eppendorf tubes and stored at -80°C until DNA and metabolite extraction (performed in an aseptic operation box). Exclusion criteria included antibiotic treatment within the previous 12 months, gastrointestinal co-morbidities, and cardiovascular disease from the time of fecal collection. The information on an individual’s basic characteristics is presented in **Table 1**. Body mass index (BMI) was measured using Inbody 720 (Biospace, Seoul, South Korea).

**Table 1:** Basic characteristic of volunteers.

Parameter	Mean (*SD*^#^)	Median (IQR^#^)
Age (years)	31.3 (6.1)	30 (23–54)
Weight (kg)	66.5 (9.0)	66 (49.5–84.8)
BMI^#^ (kg/m^2^)	22.6 (2.1)	23.1 (19.1–25.6)
Training (months)	18.1 (7.8)	18 (1–36)
Finishing time (min)	116.8 (19.4)	115 (92–160)

*^#^BMI, body mass index; SD, standard deviation; IQR, interquartile range.*

### Nutritional Data Collection

During the two sample time periods, each volunteer was given the same kind of food. Macronutrient intake information was obtained through a dietary questionnaire and calculated according to the China Food Composition (Pan, 2009). The questionnaire included 16 food groups with 39 food items (**Supplementary Table S1**). Analysis and visualization of the survey results data were achieved with Excel 2010 (Microsoft, Seattle, WA, United States) and R 3.3.3.

### High-Throughput Sequencing

Total genome DNA from the fecal samples was extracted using the CTAB/SDS method. The V3–V4 region of the bacterial 16S rRNA gene was amplified by PCR using universal primers (Forward, 5′-TCG TCG GCA GCG TCA GAT GTG TAT AAG AGA CAG CCT ACG GGN GGC WGC AG-3′; Reverse, 5′-GTC TCG TGG GCT CGG AGA TGT GTA TAA GAG ACA GGA CTA CHV GGG TAT CTA ATC C-3′). PCR reactions were performed in 30 μL reactions with 15 μL Phusion^®^ High-Fidelity PCR Master Mix (New England Biolabs), 0.2 μM forward and reverse primers, and 10 ng template DNA. Thermal cycling consisted of the initial denaturation at 98°C for 1 min; followed by 30 cycles of denaturation at 98°C for 10 s, annealing at 50°C for 30 s, elongation at 72°C for 30 s; and finally, 72°C for 5 min. High-throughput sequencing libraries were generated using an NEB Next^®^ Ultra^TM^ DNA Library Prep Kit for Illumina (NEB, United States) following the manufacturer’s recommendations, and index codes were added. Last, the library was sequenced on an Illumina HiSeq platform, and 250 bp paired-end reads were generated. Raw reads of the 16S rRNA sequences have been deposited in the NCBI SRA database under accession number SRP108097.

### LC-MS-Based Metabolism Analysis

Approximately 50 mg of feces was applied to the extraction procedure. The feces was extracted with 800 μL methanol, and 10 μL DL-o-chlorophenylalanine (2.9 mg/mL) was then added as the internal standard. After being ground, the samples were vortexed for 30 s and then centrifuged at 12,000 rpm and 4°C for 15 min. Finally, 20 μL of supernatant from each sample was transferred to a vial for LC-MS (liquid chromatography–mass spectrometry analysis). LC-MS analysis was performed on LC-Q/TOF-MS equipment (Agilent, 1290 Infinity LC, 6530 UHD and Accurate-Mass Q-TOF/MS) using a C18 column (Agilent, 100 mm × 2.1 mm, 1.8 μm). Chromatographic separation conditions: column temperature: 40°C; flow rate: 0.35 mL/min; mobile phase A: water+0.1% formic acid; mobile phase B: acetonitrile+0.1% formic acid; injection volume: 4 μL; automatic injector temperature: 4°C. MS parameters: ESI+ (positive ion mode): capillary voltage: 4 kV; sampling cone: 35 kV; source temperature: 100°C; desolvation temperature: 350°C; cone gas flow: 50 L/h; desolvation gas flow: 600 L/h; extraction cone: 4 V. ESI- (negative ion mode): capillary voltage: 3.5 kV; sampling cone: 50 kV; source temperature: 100°C; desolvation temperature: 350°C; cone gas flow: 50 L/h; desolvation gas flow: 700 L/h; extraction cone: 4 V. scan time: 0.03 s; interscan time: 0.02 s; scan range: 50–1000 m/z.

### Bioinformatics and Statistical Analysis

For the analysis of bacterial community structure, paired-end reads from the original DNA fragments were merged by using FLASH software. Paired-end reads were assigned to each sample according to the unique barcodes. 16S rRNA OTUs (operational taxonomic units) were selected from the combined reads using the QIIME software package (Quantitative Insights into Microbial Ecology). In-house Perl scripts were used to analyze alpha (within samples)- and beta (among samples)-diversity (Caporaso et al., 2010) sequences with 97% identity were assigned to the same OUT. We selected a representative sequence for each OUT and used the RDP classifier to annotate taxonomic information for each representative sequence. A metagenomic biomarker-discovery approach, linear discriminant analysis effect size (LEfSe) analysis, was performed to identify differentially abundant bacterial taxa among groups (Segata et al., 2011). Bacterial taxa with a linear discriminant analysis (LDA) score > 2 and *p* < 0.05 were considered significantly different taxa. The non-parametric Wilcoxon rank-sum test was further used to examine the differences in the relative abundance of taxa between groups with 95% confidence intervals. KEGG pathways and COG (clusters of orthologous group) functions analysis was performed with PICRUSt (phylogenetic investigation of communities by reconstruction of unobserved states) based on 16S rRNA sequencing data (Langille et al., 2013). Statistical differences (*p-*value < 0.05) were further achieved using Welch’s *t*-test in STAMP software (Parks et al., 2014).

After data mining with Mass Profiler software (Agilent), the metabolism data were normalized and edited into a two-dimensional data matrix. In total, 1206 features in the (ESI+) ion mode and 1340 features in the (ESI-) ion mode were acquired in this study. Furthermore, multivariate analysis (MVA) was performed with SIMCA-P 13.0 software (Umetrics AB, Umea, Sweden). OPLS-DA (orthogonal projections to latent structures discriminant analysis) was used to develop models to differentiate the metabolites that were altered after running a half-marathon race (Bylesjö et al., 2006). The variable importance in projection (VIP) value was produced by the OPLS-DA models in the ESI+ mode and ESI- mode (Tremaroli et al., 2009). The VIP score is a parameter that shows the importance of a variable. The variable with VIP score > 1 is considered the significant difference. Overrepresentation (enrichment) analysis of the metabolites was performed using MBRole, with the FDR (false discovery rate) corrected *p*-value (*q*-value) < 0.05 deemed to be significant (Chagoyen and Pazos, 2011).

The Spearman correlation was used to examine the correlation between GM and environmental factors, i.e., food component intake and fecal metabolites, using the R package Hmisc. Correlations with a *p*-value less than 0.05 were selected to construct the network map using Cytoscape software (Doerks et al., 2002). Redundancy analysis (RDA) and Monte Carlo Permutation Procedure (MCPP) with 999 permutations were performed using the vegan package of R.

## Results

### Basic Characteristic of Volunteers

Twenty amateur runners with an average age of 31.3 years were included in this study (**Table 1**). The average training time of the runners was 18.1 months. The completion time of the half-marathon (21.1 km) was from 92 to 160 min. The marathon race started at 8:00 am. Fecal samples before running (BEF group) were collected the morning of the marathon race (5:00–7:00 am), while samples after running (AFT group) were collected the next morning (5:00–7:00 am).

### Fecal Metabolome Analysis of the Enteric Changes in Marathon Runners

**FIGURE 1:**
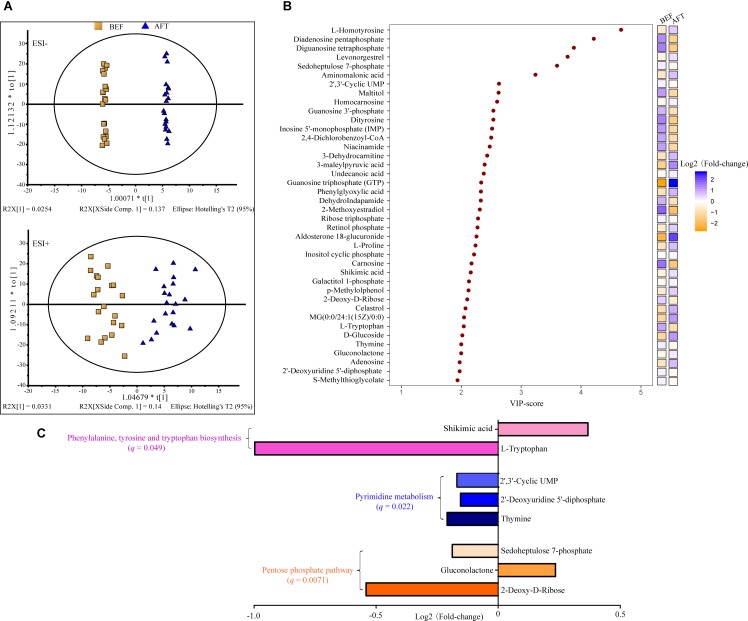
Liquid chromatography–mass spectrometry analysis (LC-MS)-based metabolomics analysis of the gut metabolome. **(A)** OPLS-DA score scatter plot obtained from LC-MS data in the ESI– and ESI+ modes. *R*^2^: the total explained variation of the model; describes how well the derived model fitted the data. *Q*^2^: the predictability of the model; provides a measure of the model quality. **(B)** Variable importance in projection (VIP) analysis based on OPLS-DA. **(C)** KEGG pathway enrichment of the significantly changed metabolites.

To address the enteric changes, metabolites extracted from feces before and after running were detected using a LC-MS-based metabolomics approach. As shown in **Figure 1A**, the metabolite concentrations of the BEF and AFT groups were compared using OPLS-DA, which was conducted to maximize the difference between the two groups. The VIP score is an indication of the contribution to the OPLS model (**Figure 1B**). Overall, 40 metabolites were significantly changed after running, with VIP scores > 1, including 9 nucleic acid components, 8 organic acids, 4 sugars, 3 alcohols, 3 steroids, 3 lipids, 3 amino acids, and 7 other metabolites. Compared to the fecal profile before running, 19 metabolites were decreased with log_2_ FC (log_2_ fold-change) less than 0, while 21 metabolites were reduced (log_2_ FC > 0). Organic acids (7 organic acids) were identified as the major elevated metabolites, while nucleic acid components (7 nucleic acid components) were the highest proportion in the reduced metabolites. L-Homotyrosine (log_2_FC = 0.6), diadenosine pentaphosphate (log_2_FC = -1.3), diguanosine tetraphosphate (log_2_FC = -1.5), levonorgestrel (log_2_FC = 0.3), and sedoheptulose 7-phosphate (log_2_FC = -0.2) were the top 5 metabolites that were changed dramatically, with VIP scores greater than 3. In addition, guanosine triphosphate (GTP), which is involved in energy metabolism for several biosynthetic pathways, was remarkably increased by a fold change of 2.6.

To identify pathways affected by running the half-marathon, 25 out of the 40 differential metabolites with a known KEGG ID were used for KEGG pathway enrichment analysis (**Figure 1C**). Ultimately, 8 metabolites were significantly (*q* < 0.05) enriched into 3 pathways: the pentose phosphate pathway, pyrimidine metabolism, and phenylalanine, tyrosine, and tryptophan biosynthesis. The pentose phosphate pathway, including 2-deoxy-d-ribose, gluconolactone and sedoheptulose 7-phosphate, was the most significantly enriched pathway, with a *q*-value of 0.0071. All 3 metabolites (thymine, 2′-deoxyuridine 5′-diphosphate, and 2′, 3′-cyclic UMP) in the pyrimidine metabolism pathway were decreased after running the half-marathon. The L-tryptophan in the phenylalanine, tyrosine and tryptophan biosynthesis pathway was the most decreased, with log_2_FC = -1.

### Responses of the GM to the Enteric Changes in Marathon Runners

We next examined whether the enteric changes after running a half-marathon could alter the composition of the gut microbial community. The fecal microbiota composition was analyzed based on high-throughput sequencing of the V3–V4 region of the 16S rDNA gene. Forty samples taken before and after running were sequenced, which yielded 2,881,919 combined reads with an average length of 411 bps. In total, 811 OTUs were obtained from the 40 samples, including 21 phyla, 34 classes, 71 orders, 123 families, 317 genera, and 564 species. According to the α-diversity analysis, our study did not reveal any significant differences in diversity after finishing a half-marathon race. Nevertheless, more special taxa at each level (from phylum to genus) were detected after running than before: 26 OTUs were specially detected in the AFT group, while 15 special OTUs were found in the BEF group (**Table 2**). At the phylum level, *Lentisphaerae* and *Acidobacteria*, whose functions in the human gut are unknown, were detected specifically after running.

**Table 2:** Group-specific taxonomic composition of GM before and after running (from phylum to genus).

Taxa	BEF-specific	AFT-specific
Phylum		*Lentisphaerae* *Acidobacteria*
Class		*Lentisphaeria* *Acidobacteriia*
Order	*Deltaproteobacteria_Incertae_ Sedis*	*Rickettsiales* *Victivallales* *Acholeplasmatales* *Subgroup_10*
Family	*Hyphomonadaceae* *Planococcaceae* *Syntrophorhabdaceae*	*NS72* *Acholeplasmataceae* *MNG7* *Mitochondria* *Victivallaceae* *JG35-K1-AG5*
Genus	*Delftia* *Ezakiella* *Lysinibacillus* *Hyphomonas* *Porphyromonas* *unclassified_f__ Porphyromonadaceae* *Selenomonas_3* *Buchnera* *Kurthia* *Syntrophorhabdus* *Leuconostoc*	*norank_f__JG35-K1-AG5* *Dethiobacter* *Bulleidia* *norank_f__MNG7* *Succinivibrio* *Victivallis* *norank_f__Veillonellaceae* *Gardnerella* *norank_f__Mitochondria* *norank_f__NS72* *unclassified_f__ Desulfovibrionaceae* *Acholeplasma*

**FIGURE 2:**
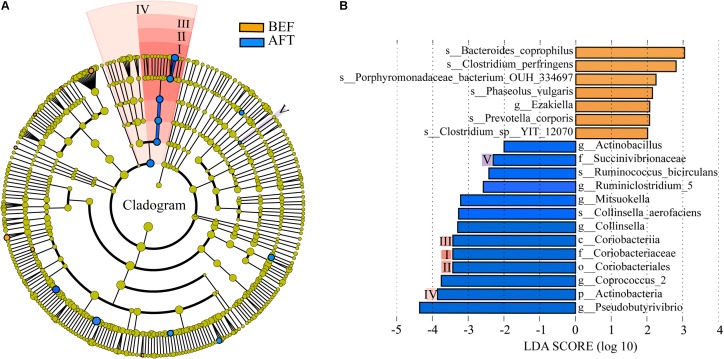
Differentially abundant bacterial taxa between the BFE and AFT groups. **(A)** Cladogram generated by LEfSe indicating differentially abundant bacterial taxa. **(B)** LDA scores for the differentially abundant bacterial taxa. Bacterial taxa significantly enriched in the BEF group (LDA score > 2) or AFT group (LDA score < –2) were detected by LEfSe analysis (*p* < 0.05). (p, phylum; c, class; o, order; f, family; g, genus; s, species).

A comparison of the relative abundance of taxa was performed by linear discriminant analysis effect size (LEfSe) analysis. LEfSe, an algorithm for high-dimensional biomarker discovery, explained the identified taxa characterizing the differences between the BEF and AFT groups (**Figures 2A,B**). The results showed that 20 bacterial clades were enriched in the AFT group, while 7 taxa were more abundant in the BEF group (*p* < 0.05). At the genus level, *Pseudobutyrivibrio*, *Coprococcus_2*, *Collinsella*, and *Mitsuokella*, which increased after running, were identified as the top 4 biomarkers, with an LDA score higher than 3.0, whereas the most enriched bacterial clade in the BEF group was *Bacteroides coprophilus*. Differentially abundant taxa from the phylum to genus level were further analyzed by Statistical Analysis of Metagenomic Profiles (STAMP) analysis using Welch’s *t*-test (**Figure 4**). STAMP analysis yielded 15 differentially abundant OTUs (*p* < 0.05), namely, 7 increased OTUs and 8 decreased OTUs, after running. The genus *Coprococcus_2* had the most increased taxa, with a fold-change of almost 3. Other significantly changed bacterial genera included *Collinsella*, *Romboutsia*, and *Pseudobutyrivibrio*.

**FIGURE 3:**
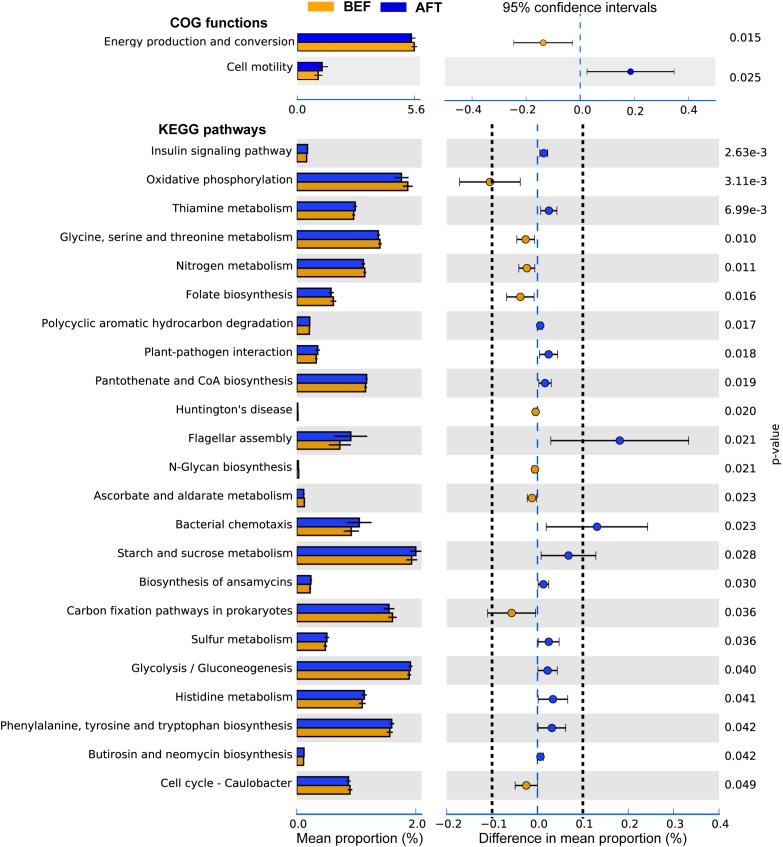
Extended error bar plot indicating the functional differences of GM in runners before and after running. COG and KEGG categories were obtained from 16s rRNA gene sequences using PICRUSt. Significant differences between two groups were tested with Welch’s *t*-test (*p*-value < 0.05). The KEGG pathways with the difference in mean proportion effect size > 0.1% were separated by dotted lines.

To determine potential functions of the GM that may be altered by an intensive running, COG functions and KEGG pathways were predicted by PICRUSt analysis (**Figure 3**). A total of 26 COG terms and 222 KEGG pathways were generated by the prediction. Comparison using Welch’s *t*-test indicated that “Cell motility” function of GM was significantly induced after running, while “Energy production and conversion” was repressed. Furthermore, 23 KEGG pathways in GM were showed significantly altered by running. Among these, “Flagellar assembly” and “Bacterial chemotaxis” pathways associated with “Cell motility” function were showed with the maximum increase in mean proportion. The down-regulated pathway with the maximum proportion was “Oxidative phosphorylation,” which was concerned in the microbial “Energy production and conversion” function.

### Correlations Between the Fecal Metabolome and Gut Microbiome

**FIGURE 4:**
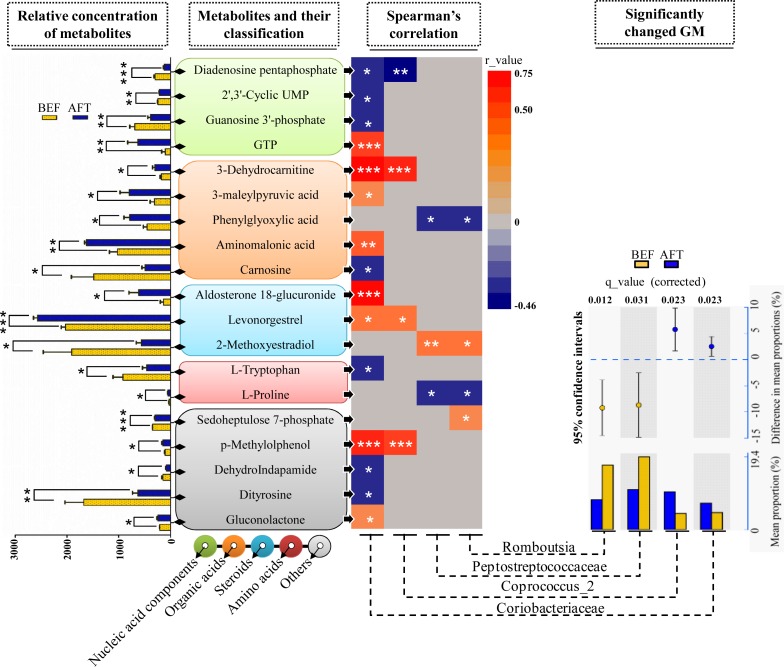
Correlation analysis of the microbiome and metabolome. Two-sample *t*-test statistics were used for the relative abundance of differential metabolites. Differentially abundant taxa as identified by STAMP analysis with 95% confidence intervals. (^∗^*p* < 0.05, ^∗∗^*p* < 0.01, ^∗∗∗^*p* < 0.001).

To address the correlations between the differential gut microbiome and the enteric changes, the Spearman correlation coefficient (*r*) was computed for the 40 differential metabolites and 9 bacterial taxa (the relative abundance was more than 3%) detected by both LEfSe and STAMP analyses (**Figure 4**). The results showed 26 statistically significant interactions between 19 metabolites and 4 bacterial taxa (*p* < 0.05). Remarkably, the increase in the bacterial family *Coriobacteriaceae* after running was shown to correlate with 15 differential metabolites: 4 nucleic acid components, 4 organic acids, 2 steroids, 1 amino acid, and 4 other metabolites. Aldosterone 18-glucuronide, 3-dehydrocarnitine, *p*-methylolphenol, and guanosine triphosphate (GTP) were found to have strong positive interactions with *Coriobacteriaceae* (*r* > 0.5, *p* < 0.001). It has been documented in previous studies that the *Coriobacteriaceae* family is involved in bile acid metabolism and that a voluntary wheel running exercise could reduce the proportion of secondary bile acids in feces. However, no significant changes in the detected 16 bile acids (**Supplementary Table S2**) between before and after running were observed. Another bacterial taxon enriched in the AFT group, the genus Coprococcus_2, was observed to have strong positive interactions with 3-dehydrocarnitine and *p*-methylolphenol (*r* > 0.5, *p* < 0.001). The *Peptostreptococcaceae* family (phylum *Bacteroidetes*) and one of its members, the genus *Romboutsia*, were shown to be weakly related to several metabolites (*r* < 0.5, *p* < 0.05).

To further identify the impact of fecal metabolites on the changes of GM, RDA was performed using six differential metabolites with their VIP scores > 3 as ‘environmental variables’ and the relative abundances of the OTUs as ‘species variables’ (**Supplementary Figure S1**). MCPP test showed that the global model was significant in the RDA (*p* = 0.038). In total, the six tested metabolites contributed only 14.52% of the changes of OTUs. Aminomalonic acid was mainly environmental variable for the observed variation of GM after running, followed by Sedoheptulose 7-phosphate and L-Homotyrosine. Remarkably, Aminomalonic acid is not from the hydrolysis of the 20 major amino acids, while it has been previously isolated from proteins of *Escherichia coli*. This suggests that the observed alterations of fecal metabolites after an intensive exercise are probably derived from GM.

### Correlations Between Food Macronutrients and Gut Microbiome

**FIGURE 5:**
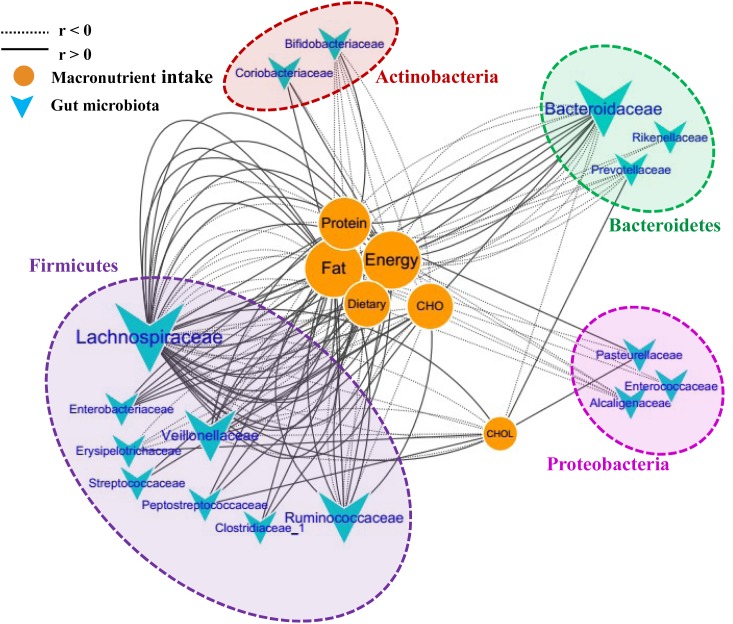
Correlation network of the GM and macronutrient intake. The network consists of 135 edges (significant correlations with *p* < 0.05) and 22 nodes (bacterial taxa and macronutrient). The bacterial taxa nodes are clustered in four subnetworks (phylum taxa). A solid line shows positive correlations; a dotted line shows negative correlations, and a wider line shows the larger correlation (absolute values of *r*). CHO, carbohydrate; CHOL, cholesterol.

To further investigate the correlations between the microbiota composition and food intake, the Spearman correlation coefficient (*r*) values of the top 50 abundant OTUs and 6 food macronutrients were determined. Sixteen bacterial taxa (family level) were shown to have 135 significant correlations with the macronutrients (*p* < 0.05). The network consists of taxonomic groups within four phyla: *Firmicutes*, *Proteobacteria*, *Bacteroidetes*, and *Actinobacteria* (**Figure 5**). Fat and energy were the largest connected components in the network, and both had 32 links (correlations) with GM. The taxonomic groups within the family *Lachnospiraceae* and fat showed strong positive correlations (*r* > 0.5, *p* < 0.001). CHO (carbohydrate) intake was strongly positively correlated with *Enterobacteriaceae*, *Veillonellaceae*, and *Clostridiaceae*, while CHOL (cholesterol) intake was strongly positive correlated with *Erysipelotrichaceae* and *Prevotellaceae*. Families *Bifidobacteriaceae* and *Alcaligenaceae* were strongly negatively correlated with these macronutrients. The significantly changed families after running, *Peptostreptococcaceae* and *Coriobacteriaceae*, were also found to correlate positively with food macronutrients. These results suggested that dietary intervention was another important factor that could alter microbial communities in a rapid manner.

## Discussion

Long-term dietary intake and exercise have been reported to influence the composition and function of the human GM. A previous study reported that short-term (1 day) diet alteration influences the composition of GM (David et al., 2014). This study firstly investigated the alterations of GM after an endurance running within a single day. The metabolic changes in the feces and the GM of 20 amateur half-marathon runners were examined using LC-MS-based metabolomic analysis and 16S-rDNA-based microbiome analysis.

Our study showed a striking metabolic change in the gut environment after finishing a half-marathon race. A total of 40 fecal metabolites were significantly changed: 19 metabolites were decreased, and 21 metabolites were increased. Seven organic acids, namely, 3-maleylpyruvic acid, phenylglyoxylic acid, 3-dehydrocarnitine, aminomalonic acid, shikimic acid, homocarnosine, and undecanoic acid, were significantly increased after running; 3-maleylpyruvic acid increased the most (FC = 1.4). Fecal organic acids were produced partly from the organic compound metabolism of the GM. 3-Maleylpyruvic acid is a product of gentisic acid oxidation by *Pseudomonas* during the metabolism of tyrosine (Sugiyama et al., 2014). It is converted to 3-fumarylpyruvate and then to fumarate and pyruvate by a variety of bacteria (Lack, 1961; Qu and Spain, 2011). Pyruvate can be converted into lactate, and the accumulation of lactate is associated with muscle fatigue in long-distance running. Shikimic acid is an important intermediate of the shikimic acid pathway in various GM (*Enterococcus*, Firmicutes, *Bifidobacterium*, and others) and plants, with very low concentrations, while human cells lack the shikimic acid pathway (Bochkov et al., 2012). Thus, products of the shikimic acid pathway, including phenylalanine, tyrosine, and tryptophan, represent the essential amino acids that must be obtained from the metabolism of GM. These increased metabolites suggest a microbiota-derived metabolism that was promoted by running. Of the 7 reduced nucleic acid components, diadenosine pentaphosphate (Ap_5_A) and diguanosine tetraphosphate (Ap_4_A) were the most differential metabolites, with VIP scores = 4. The diadenosine polyphosphates (APnAs, *n* = 3–6) released into the circulation from activated platelets were demonstrated to act as vasoconstrictors and to be involved in cardiovascular physiology and pathology. Ap5A is the most potent vasoconstricting diadenosine polyphosphate, followed by Ap6A and Ap4A. Ap4A cannot be detected with a normal oxygen supply in coronary venous blood but is increased during myocardial ischemia and reperfusion. Endurance running opens blood vessels and expands capillaries, allowing more oxygen to the muscles. The decreases in Ap_4_A and Ap_5_A in feces are probably caused by the reduction of Ap4A and Ap_5_A stored in myocardial-specific granules after endurance running (Luo et al., 2004; Song et al., 2009). The pentose phosphate pathway, a metabolic pathway parallel to glycolysis and involving the oxidation of glucose, was shown to be the most significantly enriched pathway after a half-marathon run (Kruger and von Schaewen, 2003). Marathon runners often experience “hitting the wall,” where the glycogen stores are depleted after long periods of exertion without sufficient carbohydrate consumption (De Feo et al., 2003). A low blood glucose level will lead to a high glucagon:insulin ratio, which will dramatically affect carbohydrate metabolism.

Although no significant differences in α-diversity were detected between before and after half-marathon running, we observed more specific bacterial taxa ranking from the phylum to species levels in the AFT group than in the BEF group. This finding indicated that running potentially increased the diversity of the GM. Generally, a more diverse GM means a “healthier” GM that promotes gut health and maintains essential structural, metabolic and signaling functions. Because very little is known about the role of the running-specific bacterial taxa detected in our study, our data can serve as a reference of these GM for other studies.

We observed significantly increased species richness after running in the families *Coriobacteriacea*e and *Succinivibrionaceae*. *Coriobacteriaceae* (phylum *Actinobacteria*) was reported to be involved in the metabolism of bile salts and steroids as well as the activation of dietary polyphenols in the human gut (Clavel et al., 2014). In particular, strong positive correlations between *Coriobacteriaceae* and the steroid aldosterone 18-glucuronide (*r* = 0.7, *p* < 0.001) were found in our study. Aldosterone 18-glucuronide, an important metabolite of aldosterone, performs many important functions, such as cell signaling, fuel and energy storage, and membrane integrity/stability. Aldosterone is a steroid hormone produced by the adrenal cortex of the adrenal gland to regulate sodium and potassium balance in the blood. Evidence has shown that the aldosterone level in plasma rises significantly in response to the stress of marathon running, the magnitude of which depends upon the intensity of the running (Newmark et al., 1976; Wade et al., 1985). The complex and reciprocal relationship between the GM and human body energy metabolism has been described recently (Lambert et al., 2015). During a marathon running, the total energy consumption of body is very large and metabolic demands were increased in skeletal muscle, liver, kidney, and adipose tissue within the next day or days after finishing the running. Combining with the prediction of the induced “Energy production and conversion” function of GM in PICRUSt analysis, our results suggest that long-distance running promotes the GM in regulating energy and hormone levels to maintain the electrolyte balance of the intestinal environment. At the genus level, half-marathon running reduced the abundance of fecal *Ezakiella* and *Romboutsia* but increased the abundance of *Coprococcus_2*, *Actinobacillus*, and *Ruminococcus bicirculans. Actinobacillus* species have been documented to be responsible for several distinct animal diseases, such as actinomycosis in cattle, potent septicemia in the neonatal foal, and human periodontal disease (Rycroft and Garside, 2000). Thus, the inhibition of this potential pathogen indicated an anti-inflammatory effect of exercise.

The effect of running on intestinal motility is widely recognized. Interestingly, we found here a running-induced “Cell motility” function of GM that associated with “Flagellar assembly” and “Bacterial chemotaxis” pathways. During a long-distance running, the intestinal tract is continuously subjected to mechanical impact from the moving legs, leading to loss of intestinal epithelial integrity. Several studies have reported the prevalence of increased intestinal permeability, bacterial translocation, and intestinal inflammation, predominantly of the lower tract, in marathon runners during and immediately after running. Accordingly, the observed changes of GM in this work were speculated partly due to the stimulation of damaged intestinal epithelial integrity induced by physical shock.

According to the above findings, half-marathon running induced striking changes to the gut environment, followed by a significantly changed composition of the GM. Combined analysis of the fecal metabolites and microbiome was used to obtain a global view of the dynamics of the intestinal ecosystem before and after a long-distance run. *Coriobacteriaceae* was shown to be correlated with 15 metabolites, suggesting that the metabolism of *Coriobacteriaceae* was the potential mechanism underlying the role of exercise in preventing disease and improving health outcomes. Additionally, significant correlations between food intake and GM were also found in our study. This finding was consistent with previous studies in which the influence of exercise on GM was associated with diet (Clarke et al., 2014). Different from the results of another study in which protein intake was the main driver that associated with exercise to change the GM, our study indicated that fat and energy intake might be the major factors.

Numerous studies have shown that marathon running immediately affects metabolites in blood, urine, muscles and lymph, within hours. These changes are related to the functions of the viscera, tissues, organs and immune system, such as increased immunity RAGE (receptor of advanced glycation end products), AGEs (advanced glycation end products), HMGB1 (high mobility group box 1) and ST2 (tumorigenicity 2), leucocytes, creatine kinase (CK), lactate dehydrogenase (LDH) activity, urea concentration, and ferric reducing ability of plasma and decreased erythrocytes, hemoglobin concentration, and hematocrit (Bekos et al., 2016). Thus, in addition to fecal metabolites and dietary intake, the observed differential GM in our study could be related to the metabolic changes in various body fluids induced by running.

This study provides insight into the health impacts of marathon running from the perspective of GM for the first time. Our data indicated that long-distance endurance running can immediately cause striking metabolic changes in the gut environment. The GM can rapidly respond to the altered fecal metabolites by the adjustment of certain bacterial taxa. These findings highlighted a microbiota-derived mechanism of the health-promoting benefits of exercise. Integrated analysis of fecal metabolites with microbiome structure revealed an important role of the family *Coriobacteriaceae* in regulating the intestinal environment in marathon runners. Therefore, our further research will focus on *Coriobacteriaceae* as a biomarker that links exercise with health improvement.

## Author Contributions

XZ, ZZ, and LZ developed the study concept and design, performed the data analysis. XZ, BH, WH, and CY collected the samples. BH and ZZ completed the DNA preparation and environmental genomic experiments. XZ and LZ drafted the manuscript. All authors read and approved the final manuscript.

## Conflict of Interest Statement

The authors declare that the research was conducted in the absence of any commercial or financial relationships that could be construed as a potential conflict of interest.

## Supplementary Material

FIGURE S1 Redundancy analysis (RDA) plot of the gut microbiota (GM) composition relative to fecal metabolites. Responding OTUs are indicated by black arrows. First and second ordination axes are plotted, explaining 5.04 and 9.48% of the proportion of variance, respectively.

TABLE S1 Food composition analysis of the dietary questionnaire.

TABLE S2 Bile acids detected in this study.
