# Supplementary figures and images for: Response of Gut Microbiota to Metabolite Changes Induced by Endurance Exercise
Source: Front Microbiol. 2018 Apr 20;9:765. doi: 10.3389/fmicb.2018.00765 (PMC5920010; doi:10.3389/fmicb.2018.00765)

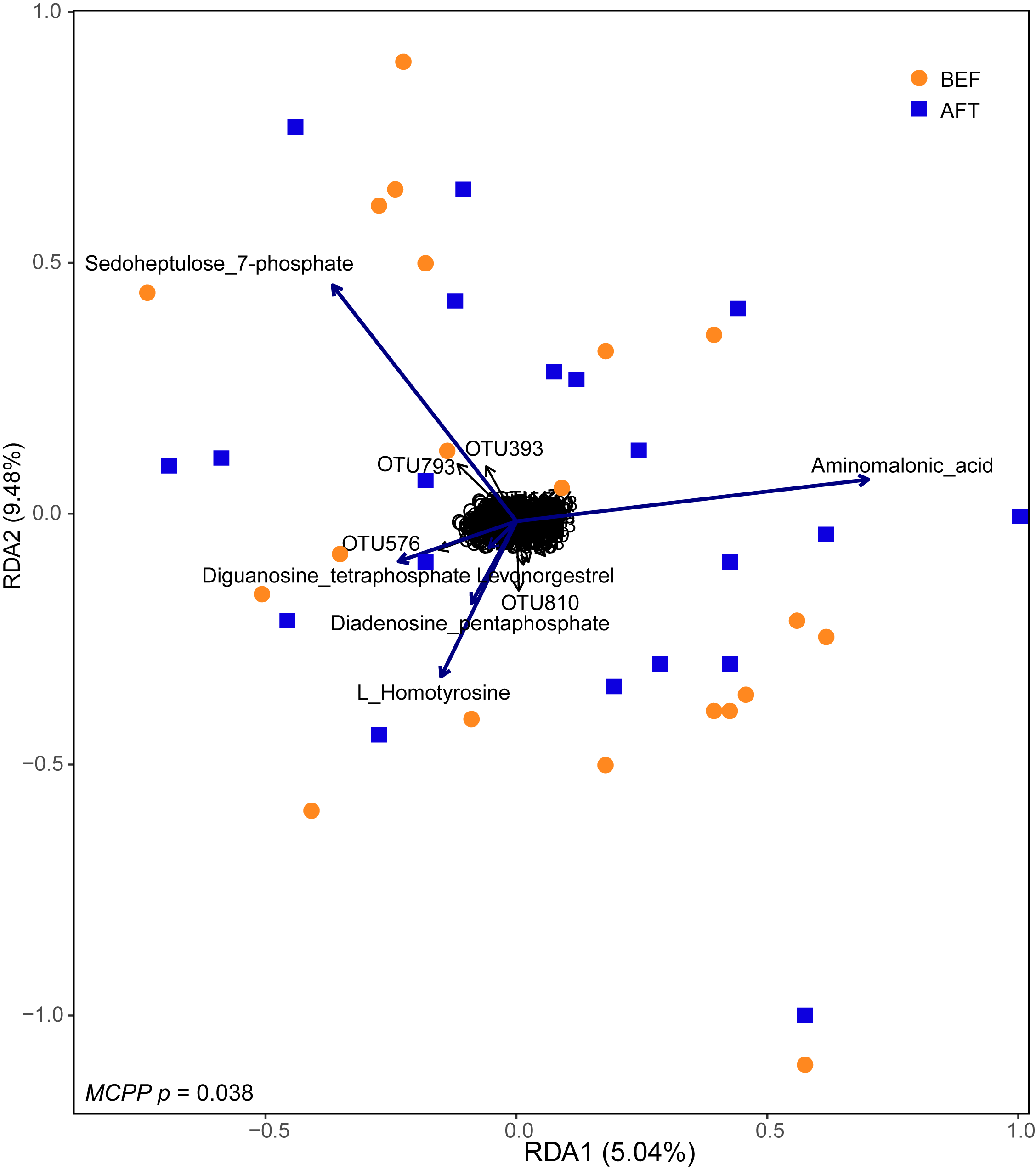

Supplement: FIGURE S1 — Redundancy analysis (RDA) plot of the gut microbiota (GM) composition relative to fecal metabolites. Responding OTUs are indicated by black arrows. First and second ordination axes are plotted, explaining 5.04 and 9.48% of the proportion of variance, respectively. [file Image_1.TIF]
